# MTA3在肺癌细胞中调控细胞凋亡机制的研究

**DOI:** 10.3779/j.issn.1009-3419.2015.10.02

**Published:** 2015-10-20

**Authors:** 海英 李, 庆苓 王, 琳 张, 海军 包, 恒 张

**Affiliations:** 221000 徐州，徐州医学院基础医学院病理教研室 Department of Pathology, College of Basic Medical Science, Xuzhou Medical College, Xuzhou 221000, China

**Keywords:** 肺肿瘤, 肿瘤转移相关基因3, 细胞凋亡, A549, H157, Lung neoplasms, Metastasis associated gene 3 (MTA3), Apoptosis, A549, H157

## Abstract

**背景与目的:**

肿瘤转移基因(metastasis associated gene, MTA)是一个肿瘤候选基因家族, 主要包括*MTA1*、*MTA2*、*MTA3*, 已有的研究证实在不同肿瘤中MTA3发挥着相反的作用, 本研究旨在探讨MTA3在肺癌细胞中调控细胞凋亡方面的影响。

**方法:**

应用Western blot方法和Real-time PCR方法检测肺癌细胞系A549和H157中MTA3的转染效率, 流式细胞仪方法检测上调/下调MTA3后肺癌细胞凋亡情况, Western blot方法检测下调MTA3后凋亡相关基因的表达。

**结果:**

在肺癌细胞系A549和H157中干扰MTA3后则促进细胞凋亡, 同时引起凋亡相关蛋白Bax、Cleved-Caspase-3、p-PARP表达上调及Bcl-2表达下调。

**结论:**

MTA3在肺癌细胞系A549和H157细胞中通过抑制凋亡相关基因的表达抑制细胞凋亡。

## Abstract (en)

**Background and objective:**

Metastasis-associated protein 3 (MTA3) was originally found as a member of a small protein family (including MTA1, MTA2 and MTA3), and it has been proven that MTA3 had different roles in different types of human cancers.The aim of this study is to explore the function of MTA3 to regulate the cell apoptosis in lung cancer.

**Methods:**

Western blot and Real-time PCR were used to detect the expression level of MTA3 after transfection in non-small cell lung cancer (NSCLC) cells A549 and H157.Apoptosis analysis was used to detect the change of cell apoptosis with upregulated/downregulated of MTA3, and Western blot was used to detect the the expression of the protein related with apoptosis, while downregulate the expression of MTA3 in NSCLC cells A549 and H157.

**Results:**

Downregulated of endogenous MTA3 could promote apoptosis in NSCLC cells, meanwhile, siMTA3 could upregulate the protein of BAX, Cleved-Caspase-3, p-PARP, and dowmregulate the protein of Bcl-2.

**Conclusion:**

The data we present here indicate that MTA3 suppress apoptosis of A549 an H157 cells by inhibiting BAX, PARP expression.

肺癌是全球发病率最高的恶性肿瘤且已经成为全球癌症死亡的主要原因之一, 并且肺癌发生率也在不同程度的增长, 而非小细胞肺癌(non-small cell lung cancer, NSCLC)占肺癌的80%左右^[1, 2]^。虽然目前手术、放疗、化疗为肺癌的主要治疗方法, 但肺癌患者的长期生存率还是比较低^[3]^, 各种基因调控、表观遗传学及微环境的影响在肿瘤发生发展和转移中起着重要的作用^[4, 5]^, 因此从肺癌发生发展的分子机制方面探讨进一步的诊疗方案显得尤为重要。

肿瘤转移基因(metastasis associated gene, MTA)是一个肿瘤候选基因家族, 主要包括*MTA1*、*MTA2*、*MTA3*, 他们分别与组蛋白脱乙酰基酶(HDAC)结合形成NuRD复合物, 使基因组蛋白发生脱乙酰化而改变其生物学行为^[6-13]^。研究报道MTA3和BCL-6在浆细胞淋巴瘤中过表达, 且负性调控促进浆细胞分化基因的表达^[14]^, 而在子宫内膜癌、乳腺癌和卵巢癌中MTA3的表达降低^[15-17]^。在乳腺癌中转染MTA3能够抑制Snail的表达, 上调E-cadherin的表达, 从而抑制上皮向间质转化^[13]^, 同时在乳腺上皮细胞中证实MTA3能够抑制Wnt4的转录活性并抑制其分泌, 下调Wnt4下游靶基因Cylin D1、TGF-3β等的表达^[18]^。近期研究证实小鼠颗粒细胞中MTA3能够促进细胞G_2_/M期转化, 从而促进细胞增殖^[19]^。在子宫非内膜样腺癌中结果显示MTA3可以作为一个独立的不良预后指标^[20]^。

研究结果显示在不同肿瘤中MTA3发挥着相反的作用, 之前的研究^[21]^发现MTA3在肺癌组织中高表达且与不良预后相关, 同时MTA3能够调控肺癌细胞增殖, 但对肺癌细胞凋亡的影响未见报道, 本研究首先在肺癌细胞中检测MTA3的转染效率, 同时应用siRNA干扰MTA3后进一步探讨MTA3在肺癌细胞系中对凋亡的影响。

## 1 材料和方法

### 1.1 主要试剂

人肺癌细胞系A549和H157购自American Type Culture Collection(Manassas, VA, USA)。细胞培养用DMEM和1640高糖培养基购自Gibco, 胎牛血清购自碧云天。干扰RNA购自上海吉玛, MTA3质粒由上海吉玛构建, 空载体为pcDNA3.1, 目的基因为pcDNA3.1-MTA3, 转染试剂购自Qiagen。RNA提取购自Invitrogen, 反转录试剂盒、Real-time PCR试剂盒及PCR引物合成购自Takara。抗BAX、c-Caspase-3、p-PARP、Bcl-2和MTA3单克隆抗体购自CST; β-actin抗体购自Santa Cruz; 辣根过氧化物酶标记的山羊抗小鼠及山羊抗兔IgG购自中杉金桥。BCA法蛋白定量试剂盒购自碧云天, 裂解液及超敏发光试剂盒购自Pierce。流式凋亡检测试剂盒购自BD。

### 1.2 细胞培养

肺癌细胞系A549和H157使用含有10%的小牛血清DMEM和1640培养基, 37 ℃、5%CO2的条件下培养, 每2天换1次液, 并用0.25%的胰蛋白酶进行消化传代。取对数生长的细胞, 转染siNC、siMTA3和PC、MTA3, 转染后48 h收集细胞^[22]^。转染序列如下:MTA3的干扰siRNA(siRNAa, 5’-CAGUGUAGAUUAUGUGCAATT-3’)及乱序阴性对照(5’-UUCUCCGAACGUGUCACGUTT-3’)。每次实验同一个处理因素设两个复孔, 重复3次实验。

### 1.3 实时定量PCR

细胞提取总RNA后, 反转录成cDNA, 使用SYBR Green法, 进行Real-time PCR扩增, 总体积20 μL。扩增过程如下:95 ℃、30 s; 95 ℃、5 s; 60 ℃、30 s, 40个循环^[23]^。β-actin作为内参。基因相对表达水平计算方式如下:ΔCt=Ct_gene_-Ct_reference_, 增加倍数用2^-ΔΔCt^方法计算。每次试验均做3个重复孔。

### 1.4 细胞凋亡检测

细胞转染siNC、siMTA3和PC、MTA3, 转染48 h后, 使用PBS清洗2次, 0.25%胰蛋白酶消化, 用培养基终止消化后, 将细胞收集到EP管中, 1, 000 rpm 4 ℃离心5 min后, 去上清。用PBS洗两遍后每个样本中加400 μL缓冲液, 吹打成单细胞悬液, 后避光加入FITC/Annexin V 10 μL和PI 5 μL染色20 min后上机检测。

### 1.5 Western blot法检测蛋白表达

收集细胞并加入裂解液充分裂解, 低温高速离心(4 ℃, 12, 000 rpm/min, 30 min), 提取上清为总蛋白。每个泳道加入总蛋白60 μg, 12%SDS-PAGE凝胶电泳, 转印(60 V, 120 min)到PVDF上。5%牛血清白蛋白室温封闭2 h^[23]^。抗BAX、c-Caspase-3、p-PARP、Bcl-2、MTA3和β-actin(1:1, 000)4 ℃孵育过夜。分别与对应的二抗(1:2, 000)室温孵育2 h, ECL显色, 结果经自动电泳凝胶成像分析仪采集, 进行灰度测定。

### 1.6 统计学方法

采用SPSS 16.0统计分析软件, 实验结果采用Mean±SD表示, 采用*t*检验进行数据分析, *P* < 0.05为差异有统计学意义。

## 2 结果

### 2.1 MTA3在肺癌细胞系A549和H157细胞中转染效率

根据之前发表研究结果选取细胞系A549和H157, 同时应用干扰效率较为明显的一对干扰序列进一步探讨MTA3对凋亡影响^[21]^。应用Western blot和Real-time PCR方法检测NSCLC细胞系A549和H157中MTA3的转染效率, 结果显示:在A549和H157细胞中干扰内源性MTA3后表达明显降低, 转染MTA3质粒后表达明显增高(图 1)。

**1:**
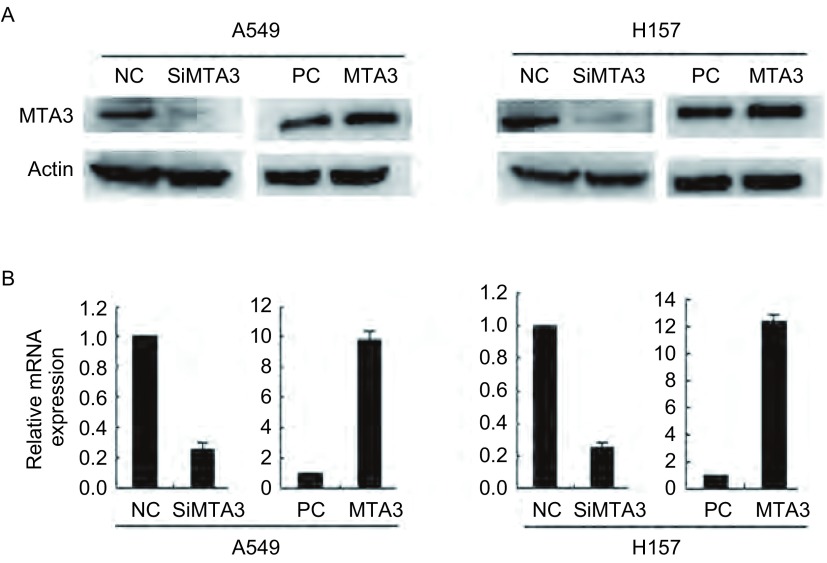
MTA3在肺癌细胞系A549和H157细胞中转染效率。A:Western blot方法检测肺癌细胞系A549和H157细胞中干扰内源性MTA3后表达降低, 上调MTA3后表达增加; B:Real-time PCR方法检测肺癌细胞系A549和H157细胞中干扰内源性MTA3后表达降低, 上调MTA3后表达增加。 Transfection efficiency of MTA3 in lung cancer cells A549 and H157.A:Western blot showed that the expression level of MTA3 was down after effect SiMTA3 and upexpression after effect MTA3 in both lung cancer cells; B:Real-time PCR showed that the expression level of MTA3 was down after effect SiMTA3 and upexpression after effect MTA3 in both lung cancer cells.NC:negative control; PC:pcDNA3.1.

### 2.2 MTA3在肺癌细胞A549和H157中对细胞凋亡的影响

在肺癌细胞系A549和H157细胞中分别上调和下调MTA3的表达, 检测其对细胞凋亡的影响, 结果如下所示:肺癌细胞系A549转染对照NC后细胞凋亡率为(9.34±0.13)%, 而干扰MTA3后细胞凋亡率为(12.81±0.39)%, 差异有统计学意义(*P* < 0.014)。在H157细胞中转染对照NC后细胞凋亡率为(12.23±0.23)%, 而干扰MTA3后细胞凋亡率为(15.83±0.16)%, 差异有统计学意义(*P* < 0.011)。同时, 在A549和H157转染MTA3上调其表达, 能够轻微抑制细胞凋亡, 但无统计学意义。可能与A549和H157细胞中内源性MTA3本身含量有关, 即使上调MTA3后其表达增加, 但也发挥不了太多的作用(图 2、图 3)。

**2:**
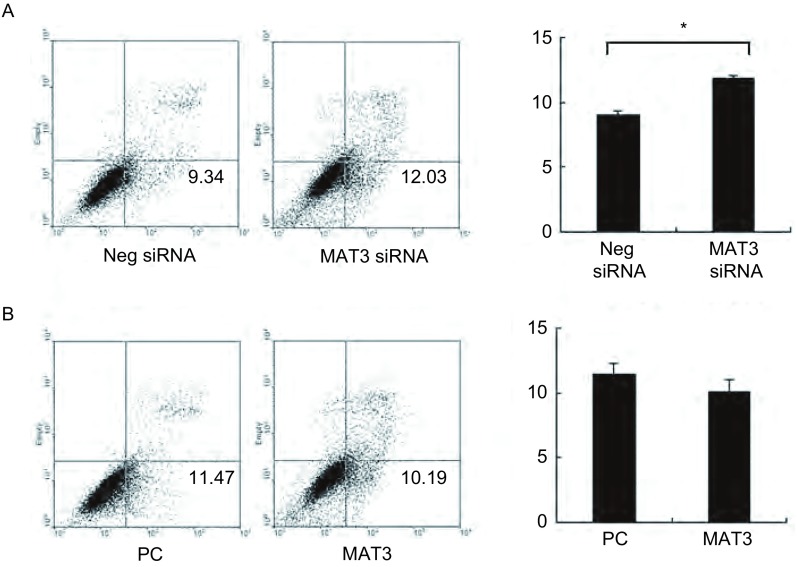
流式细胞仪检测MTA3对A549细胞凋亡的影响。A:干扰内源性MTA3后凋亡明显增加; B:转染MTA3质粒后凋亡轻微减少。 Apoptosis rate of cell was detected with tranfection of SiMTA3 and MTA3 in A549.A:Tranfection of SiMTA3 can induce cell apoptosis obviously; B:Tranfection of MTA3 can inhibit cell apoptosis mildly.^*^:*P* < 0.05.

**3:**
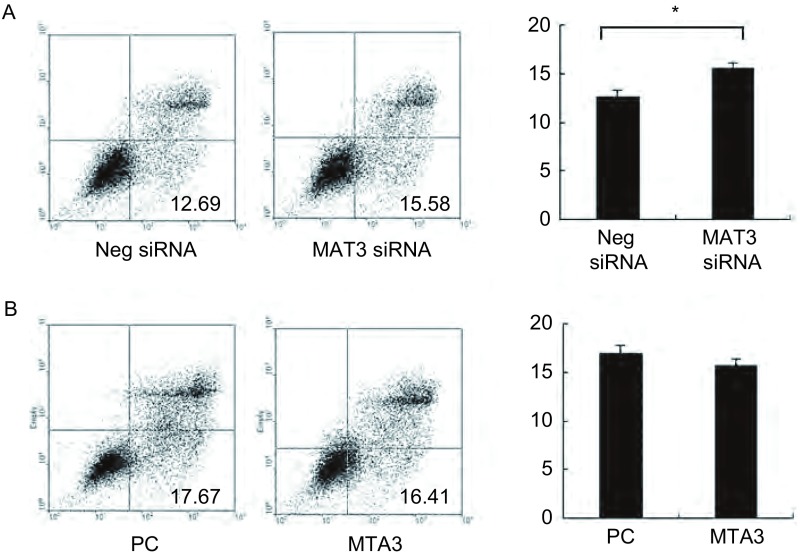
流式细胞仪检测MTA3对H157细胞凋亡的影响。A:干扰内源性MTA3后凋亡明显增加; B:转染MTA3质粒后凋亡轻微减少。 Apoptosis rate of cell was detected with tranfection of SiMTA3 and MTA3 in H157.A:Tranfection of SiMTA3 can induce cell apoptosis obviously; B:Tranfection of MTA3 can inhibit cell apoptosis mildly.^*^:*P* < 0.05.

### 2.3 MTA3在肺癌细胞系A549和H157中对凋亡相关蛋白的影响

在肺癌细胞系A549和H157中干扰MTA3后收集细胞, 提取总蛋白, 用Western blot方法检测MTA3及凋亡相关蛋白BAX、BCL-2、Cleved-Caspase-3、p-PARP等蛋白表达, 干扰MTA3后BAX、Cleved-Caspase-3、p-PARP表达明显增加, 而BCL-2的表达明显降低(图 4)。

**4:**
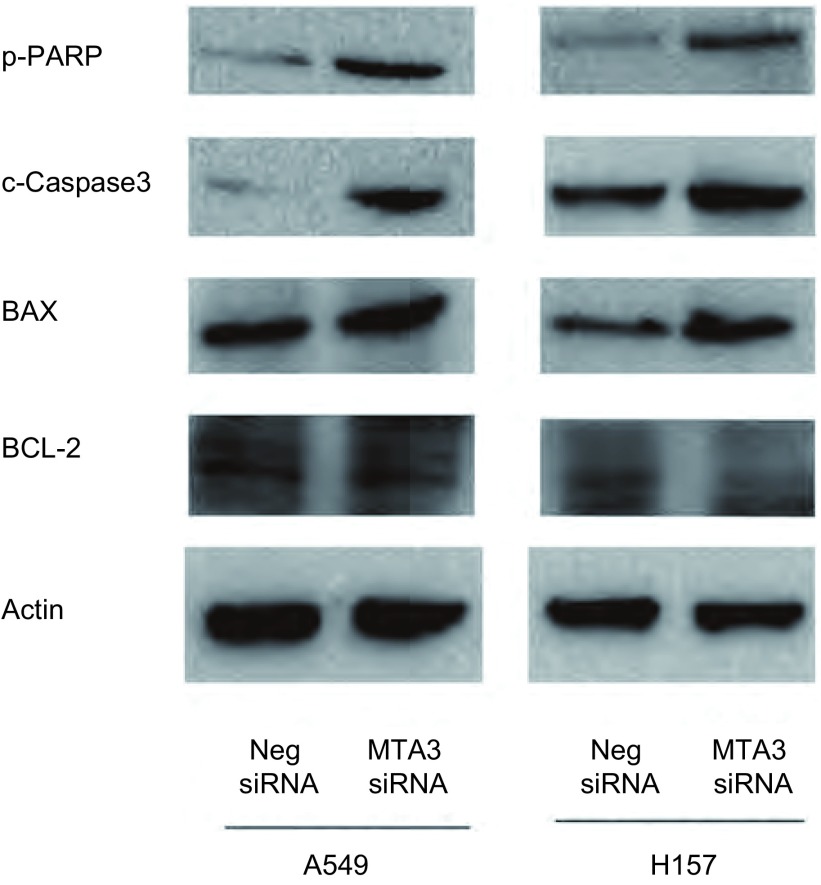
Western blot检测干扰内源性MTA3对凋亡相关蛋白的影响。在肺癌细胞系A549和H157细胞中干扰内源性MTA3后凋亡相关蛋白BAX、p-PARP、c-Caspase-3明显增加, 而Bcl-2的表达明显降低。 Expression of apoptosis related molecules levels in MTA3 depleted A549 and H157cells.Western blot analysis of a series of apoptosis related factors showed the protein levels of BAX, p-PARP, c-Caspase-3 were increased and Bcl-2 expression was decreased after silencing MTA3 in A549 and H157 cells.

## 3 讨论

NSCLC占肺癌的80%左右, 是全球发病率最高的恶性肿瘤, 已经成为肿瘤的首要死亡原因, 至今其治疗尚无突破性进展^[1, 2]^, 因此从肺癌发生发展的分子机制方面探讨进一步的诊疗方案显得尤为重要。

之前研究报道MTA3在乳腺癌、子宫内膜癌和卵巢癌中低表达^[12-14]^, 但在绒毛膜癌中高表达^[24]^, 在子宫非内膜样腺癌中研究结果显示MTA3可以作为一个独立的不良预后指标^[17]^, 我们之前的研究^[21]^发现MTA3在肺癌中的表达明显高于其癌旁正常组织, 且与不良预后有关。

在小鼠初级颗粒细胞中, 干扰内源性MTA3后Cyclin B1和Cyclin B2表达降低, 抑制细胞增殖, 而转染正义MTA3则取得相反的结果, 这一结果与我们之前的研究结果相一致, MTA3在肺癌细胞系中能够促进细胞增殖^[21]^。但是否MTA3能够进一步影响肺癌细胞的凋亡, 为了进一步验证这一观点, 我们通过上调和下调MTA3后进行细胞凋亡的检测, 发现干扰内源性MTA3后细胞凋亡明显增加。

*BCL-2*和*BAX*是凋亡相关基因, 二者通过形成复合体发挥调控细胞凋亡的作用^[25, 26]^。同时能够调控Caspase家族的活化, 而Caspase-3是Caspase家族中调控细胞凋亡的关键蛋白酶, 处于凋亡级联反应通路的核心位置, 被称为死亡蛋白酶。多种凋亡刺激因子启动不同的蛋白酶切割Caspase-3酶原, 激活Caspase-3, 活化的Caspase-3又进一步切割不同的底物, 导致蛋白酶级联反应切割放大, 最终使细胞走向凋亡^[27]^。而PARP则是最近研究发现的Caspase-3的作用底物, 活化的Caspase-3降解PARP从而促进细胞凋亡, 因此认为, Caspase-3是细胞凋亡蛋白级联反应的必经之路^[28, 29]^。我们的研究结果发现在肺癌细胞系A549和H157中干扰内源性MTA3后PARP、BAX、c-Caspase-3表达增多, 而BCL-2表达降低, 从而促进肺癌细胞凋亡。

因此我们的研究证实了MTA3在肺癌细胞系中通过调控凋亡蛋白的表达来抑制肿瘤细胞的凋亡, 初步探讨了MTA3在肺癌细胞中调控细胞凋亡的机制, 探索防治肺癌进展的新靶点。
